# Utilizing target capture sequencing to resolve the speciation history of *Echinacea* (Asteraceae)

**DOI:** 10.3389/fpls.2025.1602041

**Published:** 2025-09-09

**Authors:** Chazz Jordan, James H. Leebens-Mack

**Affiliations:** Department of Plant Biology and the Plant Center, University of Georgia, Athens, GA, United States

**Keywords:** *Echinacea*, plant conservation, phylogenomics, HybSeq, Compositae-ParaLoss-1272

## Abstract

It has been difficult to resolve relationships among many important lineages within the Asteraceae family due to interspecific hybridization and rapid species diversification throughout the history of the family. Previous efforts to resolve evolutionary relationships among *Echinacea* species have relied heavily on variation in the plastid genome with limited analysis of nuclear loci. In this study, we combine whole plastome sequences and nuclear gene capture data to reconstruct species relationships and characterize the pace of speciation across the genus *Echinacea*. With more sampling of intraspecific variation in both the plastome and nuclear sequence data, we find evidence for interspecific gene flow and reject the previously hypothesized early split between *Echinacea* lineages, including species with ranges centered in the eastern and midwestern U.S. At the same time, we find evidence for rapid radiation early in the history of *Echinacea* in agreement with previous studies. Our findings have implications for *Echinacea* conservation and trait evolution in the genus.

## Introduction

Rare plant species constitute up to a third of global plant diversity (Enquist, 2019), and they are important indicators for conservation initiatives as they can perform critical ecological functions, such as contributing to community stability (Säterberg et al., 2019). Considering the three axes of rarity defined by Rabinowitz (1981), the rarest species display highly restricted geographic distributions, low local abundance, and extreme specialization to uncommon habitats (e.g., Rabinowitz et al., 1986; Broennimann et al., 2005; Anacker et al., 2013). Comparative studies, especially those employing rigorous phylogenetics approaches, can elucidate diversification history and the processes contributing to both diversification and rarity (e.g., Molina-Venegas et al., 2020; Romeiro-Brito et al., 2023; Kress et al., 2025). For example, macroevolutionary analyses can test whether extant species diversity has been influenced by past adaptive radiations (Lunau, 2004), and reveal how the diversification of trait combinations following interspecific hybridization can spur rapid speciation (Flagel et al., 2008; Jackson et al., 2000).

**Figure 1:**
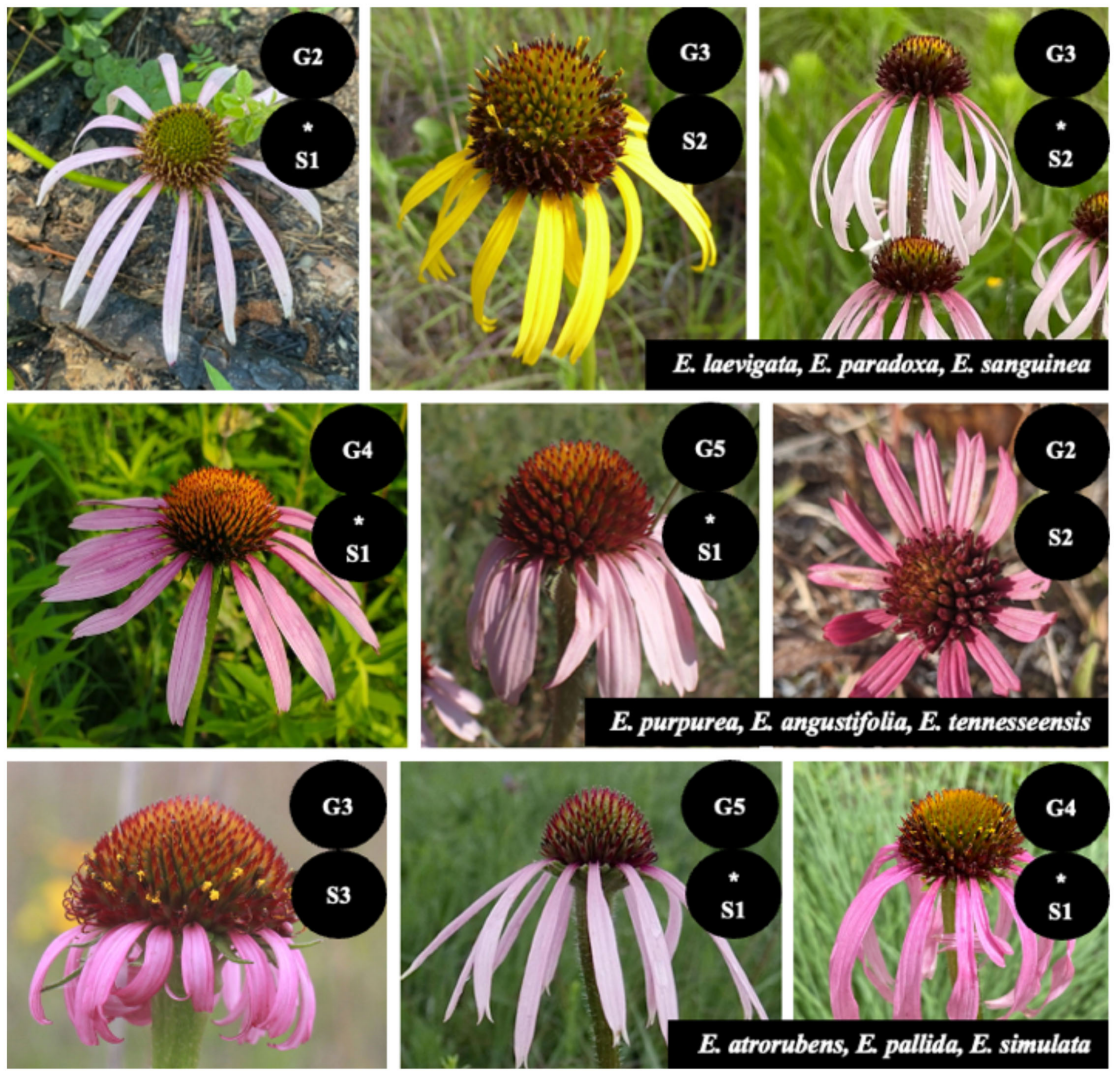
*Echinacea* species and NatureServe status rankings. All species of *Echinacea*, except for *E. purpurea*, *E. angustifolia*, and *E. pallida*, rank as G2 and G3. This indicates that most *Echinacea* species are imperiled or vulnerable. All species are ranked imperiled or critically imperiled in one or more states.

The sunflower family (Asteraceae) comprises more than 25,000 species representing 10% of all flowering plant species (Mandel et al., 2017). The evolution of the capitulum, or flower head, is hypothesized to have contributed to the hyperdiversity of Asteraceae (Mandel et al., 2019). Given its size, it is safe to say that while many common species exist within the family, a significant fraction of species likely exhibit some form of rarity. In the NatureServe database, species are described globally, nationally, and subnationally with a rank between 1 (most imperiled) to 5 (most secure). The genus *Echinacea* Moench includes nine accepted species (Plants of the World Online 2025, Flora of North America 1993+), in which, most species have bright pink and purple ray florets within capitulum heads (Kindscher, 2016; McGregor, 1968). Species range sizes vary from the highly restricted range of *E. tennesseensis* (Beadle) Small, endemic to the cedar glades of six counties in central Tennessee, to the broad range of *E. purpurea* (L.) Moench, encompassing the eastern half of the United States (McGregor, 1968). All *Echinacea* species exhibit some degree of rarity [critically imperiled (S1), imperiled (S2), or vulnerable (S3)] in at least one state within the U.S. (NatureServe), and six of the nine species in the genus are ranked as globally imperiled (G2) or vulnerable (G3) (**Figure 1**). Morphologically, *Echinacea* exhibits among-species variation in flower color, stem and leaf trichome density, capitulum size, and number per individual (Kindscher, 2016; McGregor, 1968).

It has been arduous to resolve relationships among many important lineages within the Asteraceae due to hybridization and bursts of rapid speciation throughout the history of the family (Mandel et al., 2019). Phylogenetic analyses can inform conservation efforts for rarer species exhibiting narrow species ranges, habitat specialization, and lower local abundances (e.g., Molina-Venegas et al., 2020; Romeiro-Brito et al., 2023; Kress et al., 2025). Phylogenetic analyses also contribute to taxonomic clarification and the identification of unique and recently diverged lineages, determining species value for conservation priority, as well as informing comparisons between rare and widespread species (Byrne, 2003).

Previous phylogenetic analyses of *Echinacea* have relied heavily on variation in the plastid genome. This has resulted in many relationships remaining poorly resolved (Flagel et al., 2008), even in the analyses of whole plastomes (Zhang et al., 2017). Plastid genomes have been widely utilized to resolve species relationships, but the plastid genome evolves slowly relative to genes in the nuclear genome, and the plastome is typically inherited as a single unit without recombination (Doyle, 2022). Moreover, evolving as a single locus, the evolutionary history of the plastid genome may not match the history of speciation due to incomplete lineage sorting (ILS) (Degnan and Rosenberg, 2009) and interspecific gene flow (e.g., Baldwin et al., 2023). Genome-scale multispecies coalescence analyses using many nuclear gene loci can improve the resolution of species relationships while providing insights into the pace and nature of diversification (Winter et al., 2013). Such phylogenomic investigations can also help set conservation priorities for rare endemic species (Rokas and Carroll, 2005), including *E. laevigata* (C.L.Boynton & Beadle) S.F.Blake and *E. tennesseensis* among other *Echinacea* species listed as imperiled at the state of global level (**Figure 1**).

Few studies have utilized nuclear data to resolve relationships among all *Echinacea* species, and species relationships have not been well supported in studies that have used nuclear loci AFLP markers (Still et al., 2005), *Adh* + *CesA* + *GPAT* (Flagel et al., 2008), *ITS* + *trnH – psbA* (Zhang et al., 2017), and SCoT markers (Jedrzejczyk, 2020). A robust understanding of species relationships is critical given the conservation concerns for *Echinacea* species (**Figure 1**). The primary objective of this study is to reconstruct *Echinacea* species relationships under the multispecies coalescence model and assess whether internal branch lengths in the estimated species tree imply periods of rapid speciation in the genus (Flagel et al., 2008; Zhang et al., 2017). In addition, we assess the degree of concordance between the species tree estimated using nuclear loci and the plastid genome tree. We hypothesize that our phylogenomic analyses will elucidate the evolutionary history of *Echinacea* and contribute to conservation management plans for species across the genus.

## Study system

*Echinacea* is a genus of outcrossing plants endemic to the United States, with species known for their bright floral colors, generalist pollinators, and medicinal properties (**Figure 1**; McKeown, 1999). While there is much research surrounding the medicinal and ecological attributes of *Echinacea* (Hensel et al., 2020; Manayi et al., 2015), there are fewer phylogenetic investigations of species relationships across the genus (Flagel et al., 2008; Jedrzejczyk, 2020; Zhang et al., 2017). At the same time, the relatively small size of the genus enables genus-wide investigations of *Echinacea* speciation (Flagel et al., 2008; Zhang et al., 2017). There are nine *Echinacea* species (Flagel et al., 2008, Plants of the World Online 2025, Flora of North America 1993) that vary in flower size and flower color (Wagenius and Lyon, 2010) (**Figure 1**). All *Echinacea* species, have the ability to hybridize where species ranges overlap (McGregor, 1968; Sassana et al., 2014), and rely on flying insect pollinators for reproduction (McGregor, 1968; Kindscher, 2016). Moreover, *Echinacea* populations are important components of threatened prairie ecosystems throughout the eastern half of the United States, including tallgrass, mixed grass, and short-grass prairie communities, as well as open habitats like limestone glades and hill sides (Wagenius and Lyon, 2010; Kindscher, 2016; McGregor, 1968).

## Methods

### Taxon sampling

**Figure 2:**
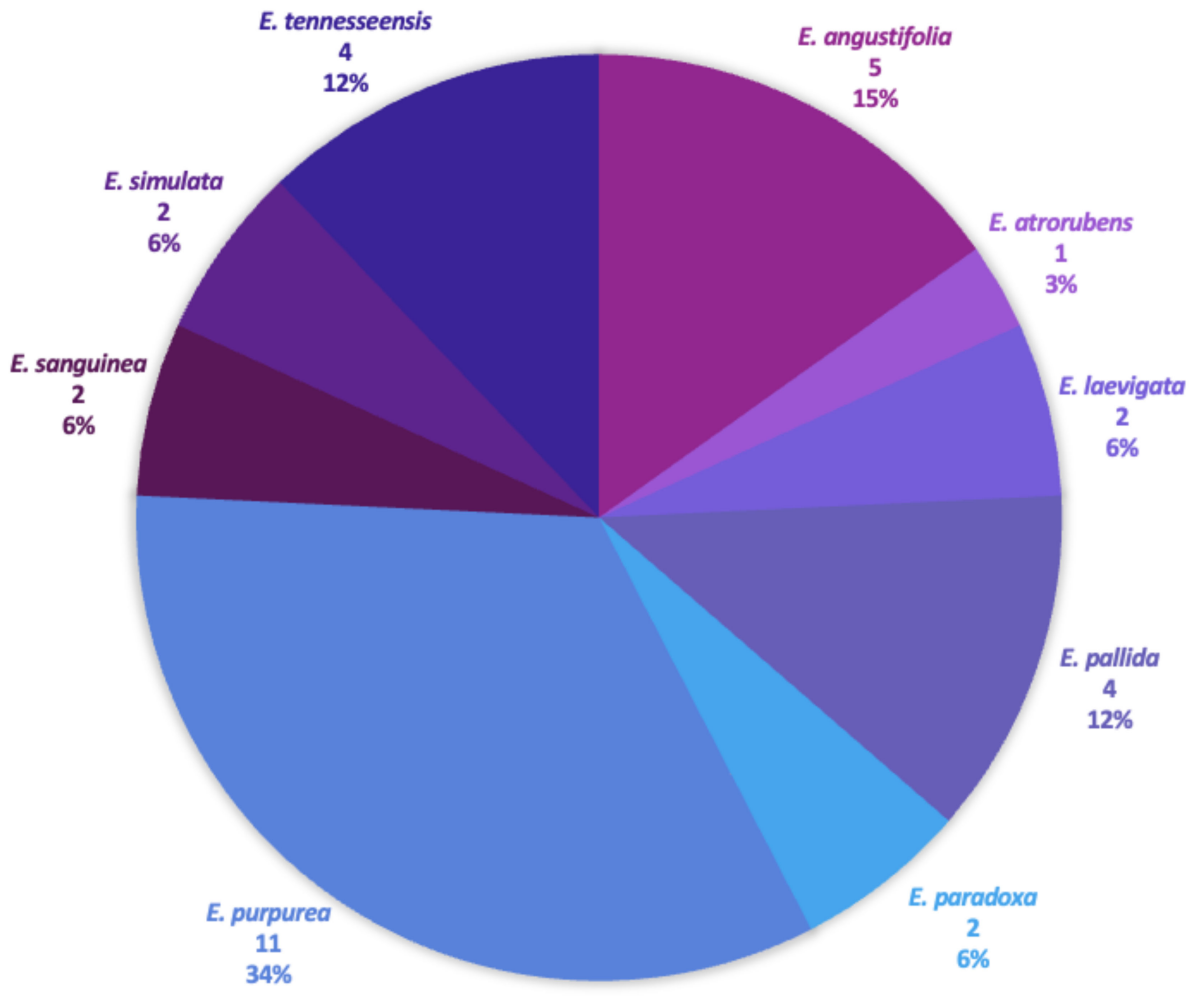
*Echinacea* study samples. Each species used in the study is displayed, as well as the number of samples per species and the species percentage used within the study.

**Figure 3:**
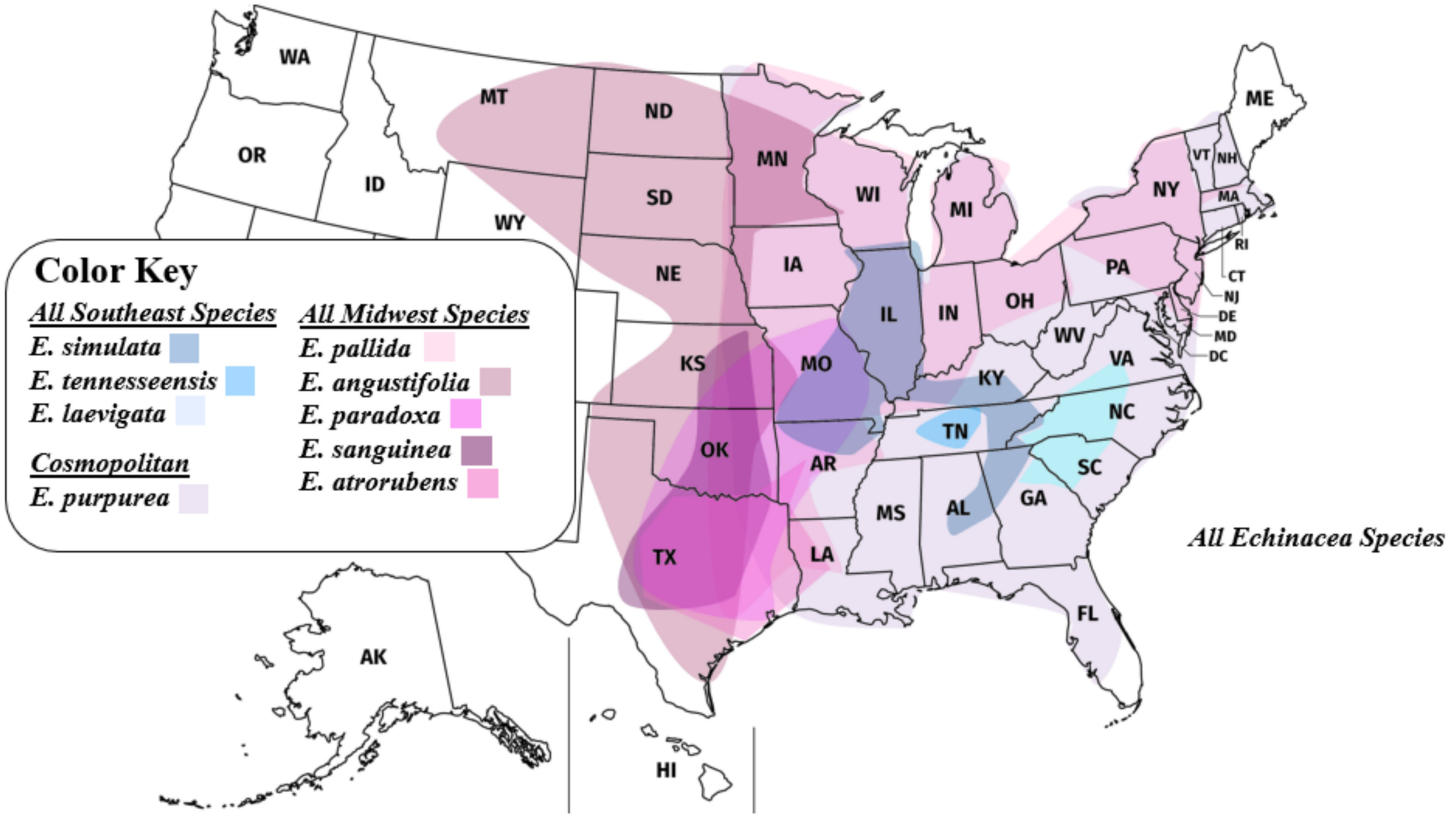
Range map of all *Echinacea* species. Southeastern *Echinacea* species (blue shades) have geographic ranges that generally lie side by side with minimal overlapping. In comparison, midwestern *Echinacea* species (pink shades) have increased overlap in their geographic ranges.

Samples for all *Echinacea* species—*E. angustifolia* DC., *E. atrorubens* (Nutt.) Nutt., *E. laevigata*, *E. pallida* (Nutt.) Nutt., *E. paradoxa* Britton, *E. purpurea*, *E. sanguinea* Nutt., *E. simulata* McGregor, and *E. tennesseensis*—were included in our phylogenomic analyses (**Figures 2**, **3**) along with available sequences for three closely related species in the Heliantheae Cassini tribe—*Helianthus annuus* L., *H. argophyllus* Torr. & A.Gray, and *Parthenium argentatum* A.Gray (Mandel et al., 2019), serving as outgroup taxa for phylogenomic analyses. Sequence data for the three outgroup samples were extracted from NCBI’s Sequence Read Archive (SRA) database (**Appendix 1**).

### DNA isolation and library creation

DNA was extracted from silica-dried, snap-frozen, and herbarium leaf tissue samples (**Appendix 1**) using QIAGEN Plant Pro Kits, and a Qubit 2.0 fluorometer broad-range assay was used to assess DNA concentrations. Shotgun sequencing libraries were constructed using Roche KAPA HyperPlus Library Kits with universal Y-yoke stub adapters (30 Mm) and dual-indexed iTru primers (Glenn et al., 2019). Double-stranded DNA was fragmented, aiming for an average fragment size between 500 and 600 bp for sequencing preparation. The DNA was then end-repaired using an A-tailing reaction for 30 min at 35°C before ligation. Universal stub adapters were then ligated to the A-tailed overhangs in an overnight incubation at 20°C, followed by a post-ligation 0.8X KAPA HyperPure bead cleanup. The cleaned ligation product was then amplified for six PCR cycles with the Roche Company KAPA HiFi HotStart Ready Mix and dual-ended primers (i5, i7), followed by a post-amplification 1x bead cleanup using KAPA HyperPure Beads (Roche, USA, Indianapolis IN) to remove free oligonucleotides and smaller fragments. Library fragment size distributions were measured using Bioanalyzer High Sensitivity DNA Kits (Agilent Technologies), and DNA concentrations (mean nM) were quantified through real-time PCR (qPCR), using KAPA Library Quantification kits and KAPA SYBR Fast qPCR Master Mix (Roche, USA, Indianapolis IN). Concentration estimates of each library were size-corrected using the following formula for the 452bp qPCR Standards:


$$x=\frac{\frac{452}{mean\;fragment\;size}}{mean\;nM}$$

They were then converted from nM to ng/μL using the following formula (660 g/mol being the approximate weight of a base pair):


$$x=\frac{660*\text{mean fragment size}*\text{size corrected nM}}{1,000,000}$$

### Hybridization and sequencing

Libraries were pooled (10–13 libraries per pool), with a target input of 150–200 ng from each library, and pools were bead-cleaned using 0.8x using KAPA HyperPure Beads (Roche). Pools were eluted from the beads in 100 μL of H_2_O and SpeedVac™ (Savant) concentrated to 7 μL as described in the Arbor Biosciences Hybridization Capture protocol. Each pool was incubated at 60°C for 24 h with 5.5 μL of the Compositae-ParaLoss-1272 target capture baits (Moore-Pollard et al., 2023). Following bait capture, hybridization reactions were amplified for 14 PCR cycles to enrich for targets, followed by a 0.8× bead cleanup for purification. Fragment size distributions for target-enriched libraries were assessed using Bioanalyzer High Sensitivity DNA chips (Agilent Technologies) and qPCR. Hybridization pools were combined for a final concentration of 10 mM for sequencing. Sequencing on the Illumina NovaSeq X Plus platform was performed through the SeqCenter (Pittsburgh, PA) sequencing service provider.

### Target sequence recovery and assembly

All Hyb-Seq reads were quality-trimmed using Fastp v.0.23.2 (Chen et al., 2018). Reads that were shorter than 21 bp after trimming were removed. The HybPiper v.2.1.6 pipeline (Johnson et al., 2016) was used to map the filtered reads to target sequences in the Compositae target file (Mandel et al., 2019) and create “supercontig” assemblies for each target locus for each sample.

### Phylogenomic analysis

Multiple sequence alignments for each locus were constructed for each target locus using MAFFT v.7.505 (Katoh and Standley, 2013) with the “—auto” flag, instructing MAFFT to choose the best alignment strategy to best fit the data. Misaligned sequences were identified and trimmed using trimAl v.1.4.1 (Capella-Gutiérrez et al., 2009). Maximum likelihood gene trees with support values were estimated using IQTree v.1.6.12 (Nguyen et al., 2015) with the “– mfp and -bb “ flags to implement ModelFinder optimization (Kalyaanamoorthy et al., 2017) and the ultrafast bootstrap (Minh et al., 2013), respectively. Species tree estimation was performed using the summary method ASTRAL v.5.7.8 (Mirarab et al., 2014) with the unrooted gene trees as input. The “-t” flag was used to report quartet scores for each node. Quartet frequencies were visualized as pie diagrams using the R packages GGTree, Ape, and TidyVerse.

### Utilizing plastome sequences from off-target reads for phylogenetic analysis

Using a plastome gene target file, plastome-encoded genes were extracted from the trimmed target-enriched sequence data as bycatch. HybPiper v.2.1.6, MAFFT v.7.505, and Trimal v.1.4.1 were used as described above to capture, assemble, and align plastome sequences. Multiple sequence alignments for 76 plastome-encoded genes were concatenated in Geneious v.2023.2.1 (Kearse et al., 2012) to construct a super matrix. A maximum likelihood tree and ultrafast bootstrap support values were estimated on the concatenated alignment using IQTree v.1.6.12 (Nguyen et al., 2015).

## Results

### Phylogenomic analysis

**Figure 4:**
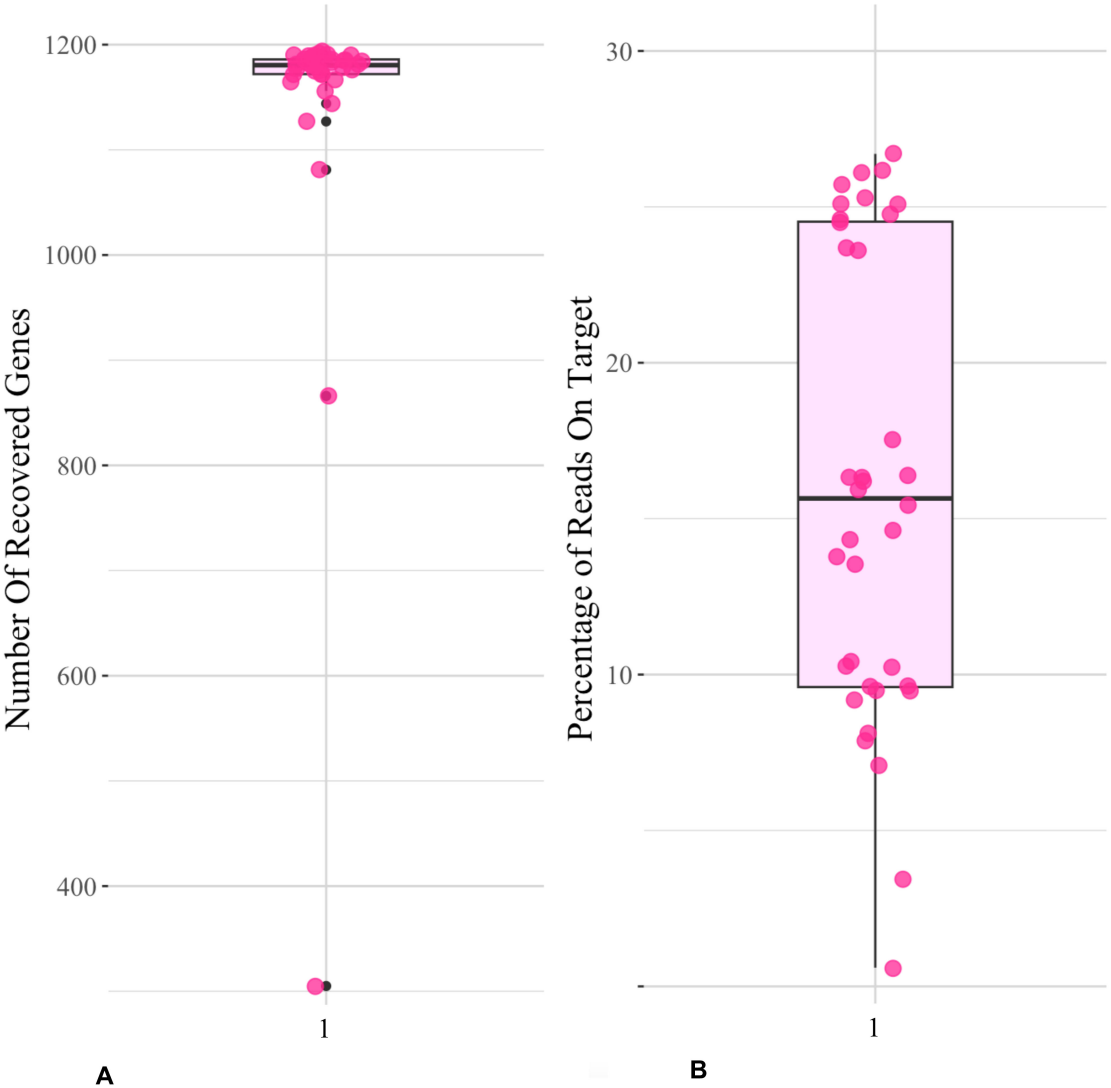
Compasitae-ParaLoss-1272 bait set on *Echinacea*. Boxplots show **(A)** the number of recovered genes, i.e., the number of targeted genes that were successfully enriched, sequenced, and assembled; and **(B)** the percentage of reads on target, i.e., the percentage of sequenced read that were successfully bound to the targeted regions of interest, which mapped to the reference target sequences from Mandel et al. (2019) using the Compositae-Paraloss-1272 baits on the *Echinacea* data set.

**Figure 5:**
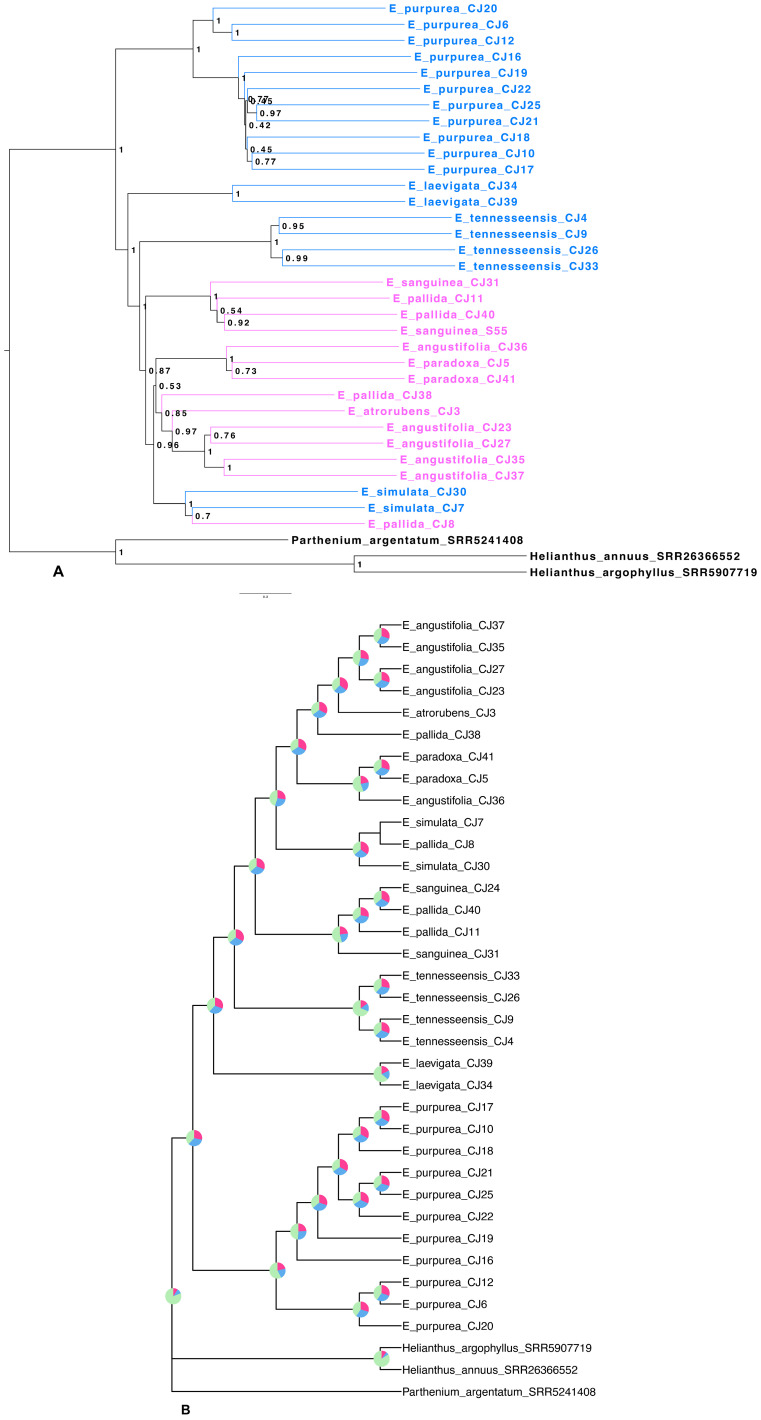
*Echinacea* species tree based on nuclear loci. **(A)** *Echinacea* species tree. Species in blue indicate southeastern species and cosmopolitan *E*. *purpurea*, while species in pink indicate midwestern species. Aside from *E*. *simulata*, eastern species are placed in a grade leading to a clade with all midwestern species. **(B)** *Echinacea* nuclear tree with quartet frequencies. Quartet frequencies on each node of the ASTRAL tree reveal extensive gene tree discordance due to rapid speciation leading to incomplete lineage sorting. For most nodes, quartets supporting alternative resolutions (Q2 and Q3) are balanced as expected with incomplete lineage sorting.

Target capture efficiency ranged between 9% and 24% for the Compositae-Paraloss-1272 bait set, resulting in 1,234–1,272 recovered genes per sample (**Figure 4**), and gene trees for all targeted loci were estimated for the ASTRAL species tree analysis. The species tree inferred from the analysis of 1,272 nuclear gene trees placed a clade of *E. purpurea* samples as sister to all other *Echinacea* species (**Figure 5**). Most species were found to be monophyletic in the species tree analysis with local posterior probabilities ranging from 0.8 to 1 (**Figure 5**). At the same time, high quartet frequencies for alternative resolutions of each node (Q2 and Q3) implicated a high degree of gene tree–species tree discordance, suggesting rapid speciation and incomplete lineage sorting, particularly along the spine of the species tree (**Figure 5**). Alternative quartet frequencies (Q2 and Q3) are generally balanced, as would be expected with ILS (**Figure 5**). Two species, *E. pallida* and *E. angustifolia*, were not recovered as monophyletic. An *E. angustifolia* individual appeared in a clade with *E. paradoxa*, while *E. pallida* samples were scattered among clades containing *E. sanguinea*, *E. atrorubens*, *E. angustifolia*, and *E. simulata* (**Figure 5**).

### Utilizing plastome sequences from off-target reads for phylogenetic analysis

**Figure 6:**
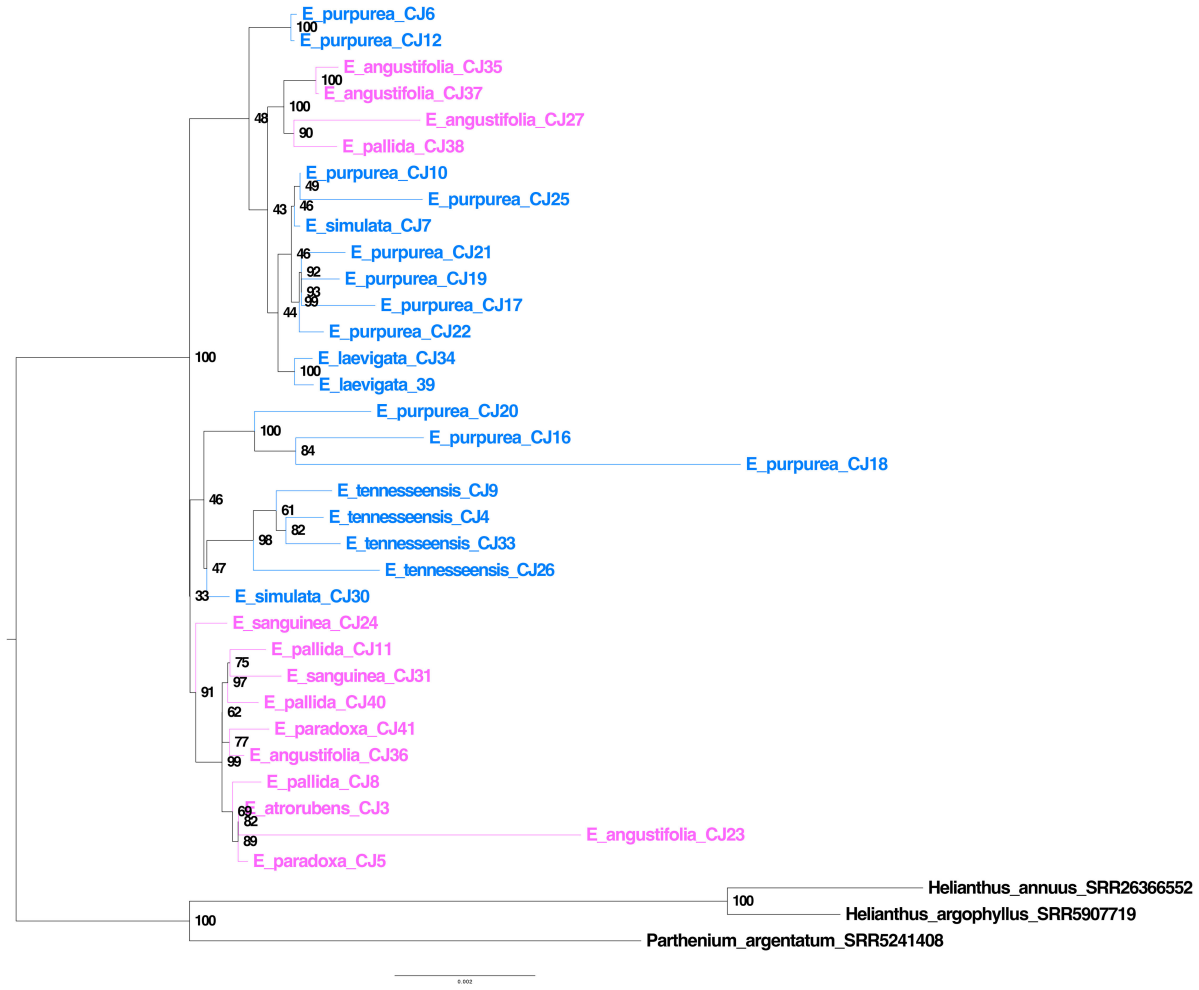
*Echinacea* plastome tree. Species in blue indicate southeastern species and cosmopolitan *E. purpurea*, while species in pink indicate midwestern species. The lack of monophyly for most species is striking, and samples do not fall into eastern and midwestern clades as found in an earlier study (Zhang et al., 2017).

Plastome genes were assembled using off-target reads from the bait capture sequence data. The plastome tree revealed extensive polyphyly for each species (**Figure 6**), and the branch lengths for internodes along the spine were very short (**Figure 6**). Most bootstrap support values along the spine of the plastome tree were low despite the massive length of the concatenated plastome gene alignment, including 67,743 bases (**Figure 6**). Interestingly, samples E_purpurea_CJ18, E_angustifolia_CJ23, and the outgroup species exhibited much longer branch lengths compared to all other ingroup samples.

## Discussion

### High gene recovery with a lower percentage of on-target sequence assembly

Here, we analyzed *Echinacea* species relationships based on an analysis of single-copy target capture genes (Mandel et al., 2019; Moore-Pollard et al., 2023). The large number of recovered genes for this genus indicates that the Compositae-Paraloss-1272 bait set is effective for sunflower family taxa beyond those originally tested (Moore-Pollard et al., 2023).

### Phylogenomic analysis shows extensive gene tree–species tree discordance

Previous plastome phylogenomic analysis with single samples per species (Zhang et al., 2017) provided a misleading view of *Echinacea* relationships and diversification. The branching patterns evident in our plastome (**Figure 6**) and ASTRAL (**Figure 5**) trees suggest that the evolutionary history of *Echinacea* includes an early burst of rapid speciation and some degree of interspecific hybridization. In contrast to the plastome tree presented by Zhang et al. (2017), our species tree (**Figure 5**) places the cosmopolitan *E. purpurea* as sister to a clade with the remainder of the genus, and most southeastern species form a grade with a clade dominated by midwestern species arising later in the evolutionary history of *Echinacea*. A rapid rate of early branching (i.e., speciation) is indicated by the short internodes along the spine of the *Echinacea* species tree (**Figure 5A**) and extensive signal for incomplete lineage sorting as seen in the high frequency of alternative quartets in the species tree analysis (**Figure 5B**). The lack of monophyly among samples of each species in the plastome tree (**Figure 6**) may also be a consequence of ILS. At the same time, polyphyly of *E. pallida* and *E. angustifolia* in both the ASTRAL (**Figure 5**) and plastome (**Figure 6**) trees implicates hybridization and interspecific gene flow.

Nearly even quartet frequencies for many nodes in the species tree estimation, shown in **Figure 5B** as pie charts, indicate high levels of gene tree–species tree discordance. Given the low posterior probabilities for many nodes on the spine of the clade dominated by midwestern species (**Figure 5A**), we cannot discount the possibility of polytomies in the *Echinacea* species tree (i.e., ancestral species spawning more than two daughter species). In support of this hypothesis, a number of nodes exhibit quartets with equal frequencies for all three resolutions of nodes on the spine of the Midwest-dominated clade (**Figure 5B**). This pattern would be expected in the face of rapid radiation and rampant ILS. At the same time, the polyphyly of some species implicates post-speciation interspecific gene flow.

*Echinacea angustifolia* and *E. pallida* are polyphyletic in the species tree based on analysis of 1272 nuclear genes. While most *E. angustifolia* samples were grouped together in the species tree (**Figure 5**), one individual was placed in a clade with *E. paradoxa*. *Echinacea pallida* samples exhibited a higher degree of polyphyly, with samples being placed in several clades with *E. sanguinea*, *E. atrorubens*, *E. angustifolia*, and *E. simulata*, all with ranges in the Midwest. Previous work has documented that all species that have overlapping ranges can hybridize (Kindscher, 2016; McGregor, 1968; McKeown, 1999). We hypothesize that more extensive range overlap among midwestern species has resulted in higher rates of introgressive hybridization. Additional sampling across the ranges of *E. simulata* and the midwestern species (**Figures 3**, **5**) is required to gain a deeper understanding of the influence of interspecific gene flow among these species.

### Plastome sequences also suggest rapid speciation and the possibility of interspecific gene flow

Most nodes on the plastome tree have bootstrap robust support values, but as seen in the species tree analysis, some nodes on the spine of the tree are poorly supported, suggesting rapid early divergence among sampled plastome lineages. Whereas Zhang et al. (2017) inferred distinct clades for eastern and western species in their whole-plastome analysis, this interpretation of the plastome history does not hold up when we sampled more than a single individual per species. Whereas Zhang et al. (2017) identified distinct eastern and midwestern species clades, our plastome tree (**Figure 6**) suggests rapid speciation early in the history of the genus, and multigene species tree estimation (**Figure 5**) suggests that *E. purpurea*, with the broadest range of all *Echinacea* species, is sister to a clade with the remainder of the genus. The lack of species monophyly in the plastome tree and the poor correspondence between the species and the plastome trees (**Figure 5** vs. **Figure 6**) are consistent with the hypothesis that species lineages retained ancestral variation in plastome haplotypes as they were diversifying. Hybridization resulting in interspecific exchange of plastomes may have also contributed to the polyphyly of the species tree (**Figure 5**) in the plastome phylogeny (**Figure 6**).

## Conclusions

The purple coneflower is known for its vibrant fluorescence and ethnobotanical significance. Being a part of the Asteraceae family, this genus has had a complex evolutionary history, including but not limited to a history of rapid radiation, variation in ploidy, and high potential for hybridization. With heavier sampling, we must reject the previously hypothesized early split between eastern and western species clades (Zhang et al., 2017). It is interesting that the most widespread species, *E. purpurea*, is sister to all other *Echinacea* species (**Figure 5**). In agreement with previous works (Flagel et al., 2008; Zhang et al., 2017), we found very short internode branch lengths along the spine of the tree, implying rapid speciation (**Figure 5**). Evidence for some interspecific gene flow is not surprising given the ability of *Echinacea* species to hybridize (McGregor, 1968) and their overlapping ranges (**Figure 1**).

Given the results of our phylogenomic analyses, we hypothesize that *Echinacea* originated in the Southeast and expanded its range into the midwest. Geographic isolation and adaptation to local environmental conditions (Baskauf et al., 1994) likely contributed to the speciation process. For example, extant *Echinacea* species exhibit variation in soil characteristics (Baskauf and Eickmeier, 1994) and climate niches (Boyd et al., 2022). The persistence of clearly distinguishable species despite the ability of all *Echinacea* species to hybridize is also suggestive of ecological divergence in the speciation process. Nonetheless, actively hybridizing populations may be of conservation concern (Sassana et al., 2014).

Phylogenetics can be used as an asset to aid conservation efforts. In the case of this study, we aimed to utilize phylogenetic inferences to improve our understanding of *Echinacea* diversity and speciation. Our findings should inform *Echinacea* conservation efforts and priorities. For example, the narrow endemic *E. tennesseensis* is phylogenetically distinct, and the polyphyly of *E. angustifolia* and *E. pallida* (**Figures 5**, **6**) implicates hybridization as a potential threat.

## Data Availability

The datasets presented in this study can be found in online repositories. The names of the repository/repositories and accession number(s) can be found in the article/**Supplementary Material**.
